# Folding Status Is Determinant over Traffic-Competence in Defining CFTR Interactors in the Endoplasmic Reticulum

**DOI:** 10.3390/cells8040353

**Published:** 2019-04-14

**Authors:** João D. Santos, Sara Canato, Ana S. Carvalho, Hugo M. Botelho, Kerman Aloria, Margarida D. Amaral, Rune Matthiesen, Andre O. Falcao, Carlos M. Farinha

**Affiliations:** 1 BioISI—Biosystems & Integrative Sciences Institute, Faculty of Sciences, University of Lisboa, Campo Grande C8, 1749-016 Lisboa, Portugal; jfsantos@fc.ul.pt (J.D.S.); sicanato@fc.ul.pt (S.C.); hmbotelho@fc.ul.pt (H.M.B.); mdamaral@fc.ul.pt (M.D.A.); aofalcao@fc.ul.pt (A.O.F.); 2 CEDOC-Chronic Diseases Research Centre, Nova Medical School, Faculdade de Ciências Médicas, Universidade Nova de Lisboa, Rua Câmara Pestana, 1150-082 Lisboa, Portugal; ascarvalho.portugal@gmail.com (A.S.C.); runem2009@gmail.com (R.M.); 3 Proteomics Core Facility-SGIKER, University of the Basque Country UPV/EHU, Barrio Sariena, 48940 Vizcaya, Spain; kerman.aloria@ehu.es; 4 LASIGE, Faculty of Sciences, University of Lisboa, Campo Grande, 1749-016 Lisbon, Portugal

**Keywords:** CFTR, endoplasmic reticulum quality control, arginine-framed tripeptides, diacidic code, interactome, folding, trafficking

## Abstract

The most common cystic fibrosis-causing mutation (F508del, present in ~85% of CF patients) leads to CFTR misfolding, which is recognized by the endoplasmic reticulum (ER) quality control (ERQC), resulting in ER retention and early degradation. It is known that CFTR exit from the ER is mediated by specific retention/sorting signals that include four arginine-framed tripeptide (AFT) retention motifs and a diacidic (DAD) exit code that controls the interaction with the COPII machinery. Here, we aim at obtaining a global view of the protein interactors that regulate CFTR exit from the ER. We used mass spectrometry-based interaction proteomics and bioinformatics analyses to identify and characterize proteins interacting with selected CFTR peptide motifs or full-length CFTR variants retained or bypassing these ERQC checkpoints. We conclude that these ERQC trafficking checkpoints rely on fundamental players in the secretory pathway, detecting key components of the protein folding machinery associated with the AFT recognition and of the trafficking machinery recognizing the diacidic code. Furthermore, a greater similarity in terms of interacting proteins is observed for variants sharing the same folding defect over those reaching the same cellular location, evidencing that folding status is dominant over ER escape in shaping the CFTR interactome.

## 1. Introduction

Cystic fibrosis (CF) is the most common lethal monogenic autosomal recessive disease among the Caucasian population, and it is caused by dysfunction of the cystic fibrosis transmembrane conductance regulator (CFTR) protein, which normally functions as a chloride/bicarbonate channel at the apical plasma membrane (PM) of epithelial cells [1]. The most common CF-causing mutation is a deletion of phenylalanine 508 (F508del) present in approximately 85% of CF patients. F508del causes CFTR misfolding, which is recognized by the endoplasmic reticulum (ER) quality control (ERQC), resulting in ER retention and targeting for degradation by the ubiquitin proteasome pathway (UPP) [2].

Understanding the exact mechanism that retains misfolded F508del-CFTR in the ER has been a major focus in the CF field. Both the folding and trafficking machineries play a relevant role in regulating ER retention and exit of CFTR [2,3], but it is not completely understood to what extent misprocessing of F508del-CFTR results from the ER retention and/or its failure in interacting with COPII export machinery. CFTR exit from the ER has been proposed to involve at least four different checkpoints, the first two of which are “folding” checkpoints, involving mainly interaction with molecular chaperones, and the last two are “trafficking” checkpoints involving recognition of trafficking/exit signals to allow its exit and incorporation into vesicles [3,4,5,6].

The first checkpoint occurs as CFTR starts to emerge from the ribosome and involves assessment of its folding state by the Hsp70 family. Very early data have shown that F508del-CFTR interacts stronger with Hsp70 machinery, with subsequent reports that degradative cochaperones such as CHIP replace the productive ones such as Hsp40 to promote the mutant’s degradation [7,8,9].

The second checkpoint is mostly dependent on CFTR glycan moieties and on interaction with the ER chaperone/lectin calnexin. At this point, CFTR folding is again assessed, and some F508del-CFTR that may have escaped the first checkpoint (and also a fraction of wt-CFTR) is targeted to glycoprotein-ER associated degradation (GERAD) [3].

The third checkpoint involves the recognition of the misfolded protein by arginine-framed tripeptides (AFTs). Mutation of these retention motifs in F508del-CFTR by replacement of arginine residues with lysine residues at positions R29, R516, R555, and R766 (termed F508del-4RK-CFTR) increases its maturation and function, although not fully to wt-CFTR levels [10]. Inactivation of two of these four AFTs, R29K and R555K, is necessary and sufficient to overcome F508del-CFTR retention, although the protein remains in a less compact structure, as determined by its slightly less resistance to proteolysis [4]. Nevertheless, the F508del-4RK-CFTR variant overcomes the ERQC without a full correction of its folding, as measured by the gating properties of the channel [5], although some reports show some correction of NBD1 by the R555K variant [11]. The F508del-4RK-CFTR variant has also been used as a tool to study the mechanism of action of small molecules, as well as other F508del-CFTR rescuing mechanisms. In fact, it was documented that treatment with the corrector VX-809 increases the amount of processed F508del-4RK-CFTR, suggesting that the compound acts by a different mechanism of the AFT abrogation, which promotes a bypass of the ERQC [12].

The fourth checkpoint occurs at the level of CFTR incorporation into COPII vesicles through recognition of a diacidic code, a specific exit code that couples cargo to the budding vesicles. The diacidic traffic code was first identified in mammalian cells and later in yeast, promoting interaction of the client proteins with the COPII machinery [13]. This motif was identified in CFTR at residues 565–567 (sequence DAD), mediating its interaction with COPII machinery, specifically with Sec23/24. Export of CFTR is highly sensitive to mutation of this highly-conserved exit code. When the aspartate residue at 567 is replaced by an alanine (D567A), CFTR processing is decreased, and its association with Sec24 is reduced [13], without however affecting the folding of CFTR [11]. Replacement of both aspartate residues with alanine (D565A, D567A, termed DD/AA-CFTR) totally blocks CFTR exit from the ER [4]. Abrogating the AFT motifs in this export-incompetent DD/AA-CFTR variant is still also ineffective at promoting ER exit, indicating that this exit motif is critical for ER export [12]. Furthermore, it was documented that VX-809 cannot overcome the export defect of the DD/AA-CFTR variant, in contrast to low temperature, which allows this variant to exit the ER through conventional ER-to-Golgi traffic [12].

Available data show that conformational alterations in F508del-CFTR are critical in determining its ER retention. CFTR interacts with many proteins that regulate its biogenesis, folding, trafficking, post-translational modifications, and function. In recent years, there has been increasing interest in defining patterns of CFTR interactions through the use of systems biology approaches, particularly proteomics. The first study assessed the interactome of wt- and F508del-CFTR in HEK293 cells and identified components of the chaperone machinery such as the Hsp90 cochaperone Aha1 [14] as differentially interacting with wt- and mutant CFTR. Recent studies have used more relevant cell models (human bronchial epithelial cells, with either endogenous or heterologous CFTR expression) and identified a subset of over 200 proteins that interact strongly with F508del-CFTR, allowing the elucidation of relevant regulatory pathways [15,16]. Similar studies have confirmed the relevance of the protein biogenesis machinery in distinguishing these two CFTR variants, through identification of the eukaryotic translation initiation factor 3 [17] as a stronger interactor of F508del-CFTR whose modulation can rescue the mutant protein to the cell surface. Most of these studies however were focused on the wt- and F508del-CFTR interactomes, although recent work also compared the F508del-CFTR interactome with that of the F508del-4RK-CFTR variant, identifying critical differential interactions, which when modulated can lead to the rescue of F508del-CFTR to the PM [18] or others focused on the interactomes of other disease-occurring CFTR variants.

Here, we aimed at exploring CFTR ERQC trafficking checkpoints by performing global comparisons of the interactions that regulate CFTR exit from the ER to identify protein factors involved in these processes. Using protein interaction profiling and global bioinformatics analyses, we identified proteins that interact specifically with peptides harboring the normal (wt) and 4RK AFT sequences, as well as proteins that interact with CFTR with/without abrogation of the DAD code. We performed an extended comparative analysis of the protein interactors identified for CFTR variants and peptide motifs and identified higher similarities for CFTR variants sharing the same folding status over those sharing the same location, revealing that the folding status is apparently dominant over the cellular localization of CFTR in shaping its local interactome.

## 2. Materials and Methods

### 2.1. Generation of Cell Lines

CFBE41o− (cystic fibrosis bronchial epithelial) cells stably expressing DD/AA-CFTR were generated as previously described [18]. Briefly, CFTR cDNA bearing D565A and D567A (DD/AA) was introduced, cloned into the lentiviral expression vector pLVX-Puro by homologous recombination using the In-Fusion HD Cloning Kit (Clontech-Takara, #121416, Saint-Germain-en-Laye, France). For lentiviral particles’ production, plasmids with the correct sequence were used to calcium phosphate-transfect HEK 293T cell. Lentiviral particles were collected 48 h after transfection and were used to transduce parental CFBE41o− cells [18].

CFBE41o− cells expressing double-tagged mCherry-FLAG-CFTR (wt, F508del, F508del-4RK or DD/AA) under a doxycycline inducible promoter were grown in DMEM with 4.5 g/L glucose and l-glutamine supplemented with 10% (*v*/*v*) FBS, 2 µg/mL puromycin, and 10 µg/mL blasticidin (InvivoGen, #ant-bl, Toulouse, France). CFTR expression was induced with doxycycline 1 µg/mL (Sigma #9891, Gillingham, Dorset, UK) (24-h treatment for wt-CFTR and 48-h treatment for CFTR mutants). All cell lines were grown at 37 °C in 5% CO_2_. CFTR levels were measured in each cell using the fluorescence quantification to determine total CFTR, given by the mCherry fluorescence intensity and PM CFTR, given by Cy5 fluorescence intensity [19].

### 2.2. Cell Lines and Compound Treatment

Parental CFBE41o− (cystic fibrosis bronchial epithelial) cells and CFBE stably transduced with wt-CFTR, F508del-, DD/AA-, or F508del-4RK (R29K, R516K, R555K, R766K)-CFTR were grown in minimum essential media with l-glutamine–EMEM (Lonza, #BE12-611F, Basel, Switzerland) supplemented with 10% (*v*/*v*) fetal bovine serum (FBS) (GIBCO^®^ Life Technologies, #10270-106, Carlsbad, CA, USA), and 5 µg/mL puromycin (50 mg/mL, Sigma, #P8833). CFBE41o− parental cells were originally derived from a homozygous F508del-CFTR patient, but have no detectable expression of CFTR. All cell lines were grown at 37 °C in 5% CO_2_. When applicable, cells were incubated with the corrector VX-809 or vehicle control (DMSO), 3 µM for 24 h or 48 h in the appropriate media supplemented with 0.1%(*v*/*v*) FBS.

### 2.3. Western Blot

Protein extracts from CFBE41o− cell lines expressing CFTR were separated on 7% (*w*/*v*) polyacrylamide gels, transferred to PVDF membranes, and analyzed by Western blot (WB). Membranes were blocked and probed with 1:5000 mouse anti-CFTR 596 (CFF, Cat No. A4) or with 1:3000 mouse anti-calnexin (BD Transduction Laboratories™, Cat No. 610523, San Jose, CA, USA), which were used as the loading control. Membranes were washed and incubated for 1 h with anti-mouse secondary antibody. Chemiluminescent detection was performed using the Clarity™ Western ECL substrate (BioRad, #1705061, Hercules, CA, USA) and the Chemidoc™ XRS system (BioRad). The quantification of band intensity was performed using the Image Lab software (BioRad) and normalized to the loading control as appropriate.

### 2.4. CFTR Immunoprecipitation

Full-length CFTR was immunoprecipitated from whole-cell lysates from CFBE41o− cells stably expressing wt-, F508del-, F508del-4RK-CFTR, or DD/AA-CFTR. Parental CFBE41o− cells were used to define the background using the anti-CFTR monoclonal antibody 596 coupled with rProtein G agarose beads. For the coupling antibody-beads, the anti-CFTR 596 was incubated with beads at a final concentration of 1 µg/mL at RT for 1 h with rocking. Beads were washed with 10 volumes of sodium borate (0.1 M) pH 9 and resuspended again in 10 volumes of sodium borate (0.1 M) pH 9 with dimethyl pimelimidate 2 HCl (DMP) (Thermo Scientific, #21667, Waltham, MA, USA) at the final concentration of 20 mM. The DMP and antibody bound-beads were mixed for 30 min at RT. The reaction was terminated by washing the beads once in ethanolamine (0.2 M) pH 8 and twice in PBS. Finally, beads were resuspended in PBS with 0.02% (*w*/*v*) sodium azide and stored at 4 °C.

For in vivo cross-linking, immunoprecipitations were performed in the presence of cells incubated with the cleavable chemical cross-linker dithiobis(succinimidyl propionate) (DSP) (Thermo Scientific, #22585) prior to lysis.

To isolate proteins interacting with CFTR AFT sequence motifs, 10-aminoacid long synthetic peptides conjugated with agarose beads (ProteoChem™, g4101, Hurricane, UT, USA), and containing the CFTR sequence around each of the AFTs, either the wild-type sequence (non-mutated AFTs: R29–CKGYRQRLEL, R516–CDEYRYRSVI, R555–CGGQRARISL and R766–CLQARRRQSV) or the Arg-to-Lys substituted versions (mutated AFTs: K29–CKGYKQRLEL, K516–CDEYKYRSV, K555–CGGQRAKISL, and K766–CLQARRKQSV) were custom-synthesized by CASLO ApS (Kongens Lyngby, Denmark). The pull-down was performed using either a mixture of the non-mutated peptide-conjugated beads or of the mutated peptide-conjugated beads in lysates from CFBE parental cells.

For the preparation of cell lysate for immunoprecipitation or peptide pull-down, cells were washed in cold PBS supplemented with 0.9 mM CaCl_2_, 0.5 mM MgCl_2_, pH 7.2, incubated for 30 min at 4 °C in either Triton lysis buffer (TBS) (25 mM Tris-HCl pH 7.4, 150 mM NaCl_2_, 1% (*v*/*v*) Triton-X 100, supplemented with protease inhibitors) in the case of full-length CFTR immunoprecipitation or PD-buffer (Tris-HCl 50 mM pH 7.4; NaCl_2_ 100 mM; glycerol 10% (*v*/*v*); NP40 1% (*v*/*v*) supplemented with protease inhibitors) for peptide pull-down, scraped, and pelleted. The supernatant from the cell lysate was pre-cleared for 1 h at 4 °C with agarose beads, followed by incubation with anti-CFTR 596 antibody cross-linked to rProtein G agarose beads or peptide-conjugated beads overnight at 4 °C. Finally, the sample was washed twice with wash buffer (Tris-HCl 100 mM; NaCl_2_ 300 mM) supplemented with 1% (*v*/*v*) Triton-X100 and twice with wash buffer without Triton. Proteins were eluted in DTT (50 mM) for 15 min at RT with rocking, followed by incubation for 5 min at 37 °C with Tris-HCl 90 mM pH 7.2, or in the case of peptide pull-down, proteins were eluted in glycine (50 Mm) for 15 min at RT followed by incubation with Tris-HCl (90 mM pH 7.2) 5 min at 37 °C.

### 2.5. Sample Preparation for LC-MS/MS

Following CFTR immunoprecipitation, complexes eluted in a solution containing DTT were loaded into filtering columns and washed exhaustively with urea (8 M) in HEPES buffer. After alkylation with iodoacetamide, proteins were incubated overnight with sequencing-grade trypsin (Promega, Madison, WI, USA). Peptides were desalted and analyzed by mass spectrometry. In the case of peptide pull-down, the eluted proteins were loaded into filtering columns and washed with 8 M urea in HEPES buffer. The sample incubated with DTT was used for reduction and then with iodoacetamide for alkylation. Proteins were incubated overnight with sequencing-grade trypsin (Promega). Peptides were subjected to mass spectrometry analysis.

### 2.6. Collection and Analysis of Peptide Spectra Using LC-MS/MS

Peptides generated as described above were desalted and concentrated [20] prior to analysis by nano LC-MS/MS using a Q-Exactive (Thermo, San Jose, CA, USA) mass spectrometer coupled to an EASY-nLC 1000 liquid chromatography system (Thermo) via a Nanospray Flex Ion Source. Peptides were loaded onto an Acclaim PepMap100 pre-column (75 µm × 2 cm, Thermo) connected to an Acclaim PepMap RSLC (50 µm × 15 cm, Thermo) analytical column. Peptides were eluted from the column using a linear gradient of 3–30% acetonitrile in 0.1% formic acid at a flow rate of 300 nL min^−1^ over 90 min (120 min for the peptide pull-down). The mass spectrometer was operated in positive ion mode. Full MS scans were acquired from *m/z* 350–2000 (1850 for peptide pull-down data) with a resolution of 70,000 at *m/z* 200. The ten most intense ions were fragmented by higher energy C-trap dissociation with normalized collision energy of 28, and MS/MS spectra were recorded with a resolution of 17,500 at *m/z* 200. The maximum ion injection time was 120 ms for both survey and MS/MS scans, whereas AGC target values of 3 × 10^6^ and 5 × 10^5^ were used for survey and MS/MS scans, respectively. In order to avoid repeat sequencing of peptides, dynamic exclusion was applied for 30 s. Singly-charged ions or ions with an unassigned charge state were also excluded from MS/MS. Data were acquired using Xcalibur software (version 4.0, Thermo).

The intensity-based absolute quantification (iBAQ) [21], a label-free proteome quantification method, was used to determine the stoichiometries of the identified proteins. This quantification was based on peak intensity in survey scans, corresponding to the sum of all the peptides intensities divided by the number of observable peptides of a protein and was integrated into the quantitative proteome software package MaxQuant [22,23]. All data were searched with Virtual Expert Mass Spectrometrist (VEMS) [24] and MaxQuant [25]. Mass accuracy was set to 5 ppm for peptides and 10 mDa for peptide fragments. Gaussian weight for fragment ions was set to 5, and the six most intense fragment ions per 100 Da were used for scoring fragment ions. Two missed cleavages were specified, and the Human Database from UniProtKB (Release 2015_02) was used, including permutated protein sequences, leaving Lys and Arg in place, together with common contaminants such as human keratins, bovine serum proteins, and proteases. The total number of protein entries searched was 136,314. Fixed modification of carbamidomethyl cysteine was included in the search parameters. A list of 8 variable modifications was considered for all data against the full protein database. Protein N-terminal Met-loss was not specified for VEMS searches since VEMS by default checks N-terminal Met-loss. The false discovery rate (FDR) for protein identification was set to 1% for peptide and protein identifications unless otherwise specified. No restriction was applied for minimal peptide length. Identified proteins were divided into evidence groups as defined previously [26]. Statistical analysis and data filtering were performed using the R statistical programming language (https://www.r-project.org/).

After identification and quantification of the mass spectrometry results, each protein was normalized to the total amount of protein in the respective sample (iBAQ quantitation). To filter for unspecific interactions, a combination test was performed between each sample and the background (pull-down from parental CFBE41o− cells) using an R script. For this, the iBAQ values for each protein from each sample replicate in the study (wt-CFTR or DD/AA-CFTR) were divided by the iBAQ values for the same protein in each replicate of the control (parental cells), resulting in nine combinations according to the number of replicates. In the case of peptide pull-down, iBAQ values for each replicate in the study were divided by the iBAQ values for the same protein in each replicate of the control (non-conjugated beads). Then, the median for the sample replicates resulting from the combination test was calculated. The affinity of a given protein for each CFTR variant was determined by the base 2 logarithm (log_2_) of the ratio in which the median for each protein in one CFTR variant (or peptide) was divided by the median for the same protein in another CFTR variant (or peptide).

A more stringent method was used for the holistic approach (full pairwise comparisons and principal component analysis (PCA)). Each protein was normalized for the total amount of proteins identified for all samples and replicates. A log_2_ for each protein was calculated, and unspecific interactions were filtered by combination between each sample and the background using an R script. The log_2_ values for each protein from each sample replicate in the study (wt-CFTR, F508del-, F508del-4RK-CFTR, or DD/AA-CFTR) were subtracted by the log_2_ values for the same protein in each replicate of the control (parental cells) resulting in nine to twelve combinations according to the number of replicates (3 or 4). In the case of peptide pull-down, log_2_ values for each protein from each sample replicate in the study (non-mutated AFTs or mutated AFTs peptides) were subtracted from the log_2_ values for the same protein in each replicate of the control (non-conjugated beads). Then, the median for the sample replicates resulting from the combination test was calculated. The affinity of a given protein for each CFTR variant was determined by subtracting the median for each protein in one CFTR variant (or peptide) by the median for the same protein in another CFTR variant (or peptide).

### 2.7. Networks and Gene Ontology

The Database for Annotation, Visualization and Integrated Discovery (DAVID) (https://david.ncifcrf.gov/) [27,28] was used to analyze the obtained dataset and identify the Gene Ontology (GO) terms that were enriched in our data. The GO terms considered were the biologic process (BP) and cellular component (CC) using Version 6.8. Networks were generated using the Agile Protein Interactome DataServed (APID) (http://cicblade.dep.usal.es:8080/APID/init.action) [29], accessed and visualized/analyzed using Cytoscape (http://cytoscape.org) [30]. Networks were presented with only the proteins detected in the interactomes. The interacting proteins were plotted according to the yFiles Organic Layout algorithm.

### 2.8. Principal Component Analysis

Principal component analysis was performed using the package “pca3d” (https://cran.r-project.org/web/packages/pca3d/index.html) within R [31]. The median of the processed immunoprecipitation values for each CFTR variant was computed for each protein; thus, a data frame with 5 columns and as any rows as the proteins measured was created. The principal components for this dataset were computed, and a 3D scatter plot of the 3 main components was used for visual inspection and clustering analysis. Due to the nature of the data provenance being the same, it was not scaled, nor centered for PCA analysis. Clustering was performed using hierarchical agglomerative clustering through the standard R function “hclust” and using average linkage as a clustering method. The resulting clusters were visually assessed using the 3D plots for different partition thresholds that produced different clustering arrangements, ranging from 3–12. It was apparent that a partition into 8 clusters produced the clearest differentiation.

### 2.9. Statistical Analysis

Two-tailed Student’s *t*-tests were performed to quantify statistical significance versus the corresponding negative control. In these analyses, *p* < 0.05 was considered as significant.

The permutation test was performed to quantify the relationship between statistical significance (−log_10_(*p*-value)), and the log_2_ ratio (protein ration between two conditions) of each protein was obtained by a volcano plot. The significance was calculated by a permutation test using a custom R script. Thus, we performed random permutations for each protein present in both samples in comparison using 10,000 permutations. Proteins were considered to have different affinity for *p*-value < 0.025. In the volcano plot, this permutation’s *p*-value is shown as −log_10_(*p*-value) and considered statistical different if −log_10_(*p*-value) > 1.60.

The Jaccard similarity index between two sets of proteins was calculated as the size of intersection of the two lists divided by the size of the union of the two lists.

## 3. Results

### 3.1. Trafficking Factors Acting at the Level of the Diacidic Exit Code

To expand previous knowledge on the ERQC for CFTR, we aimed at identifying potential new traffic factors that interact with CFTR at the ER. We focused on proteins that regulate CFTR retention/export at the fourth ERQC checkpoint [12].

To this end, we generated a novel CFBE41o− cell line stably expressing the DD/AA-CFTR variant. This novel cell line was characterized in terms of protein expression and response to modulators (Figure S1), and results showed that it recapitulated the previous findings for DD/AA-CFTR expressed in BHK cells, namely: the absence of mature CFTR (band C) and the lack of response to VX-809. We also detected an increased amount of DD/AA-CFTR when compared to F508del-CFTR (using a double-tagged mCherry/FLAG version), thus excluding possible misfolding induced by this variant (Figure S1).

We then used this cellular system in parallel with wt-CFTR CFBE-expressing cells [32] to obtain pulled-down proteins that interact with either wt- or DD/AA-CFTR following a general strategy consisting of: (i) cell lysis and immunoprecipitation (IP) of CFTR and interacting proteins; (ii) identification of proteins by LC-MS/MS; and (iii) analysis of the CFTR interactome (database search and statistical filtering). Parental CFBE cells were used as the control (background sample).

By this analysis, we identified a total of 1150 different proteins: 979 proteins for DD/AA-CFTR IP and 945 proteins for wt-CFTR IP (Datasets S1A,B, respectively), 45% being common to both sets. The analysis was performed in triplicate; similarity analysis among replicates showed an overlap of 77% in DD/AA-CFTR and 62% in wt-CFTR, confirming the reproducibility of the approach.

**Figure 1:**
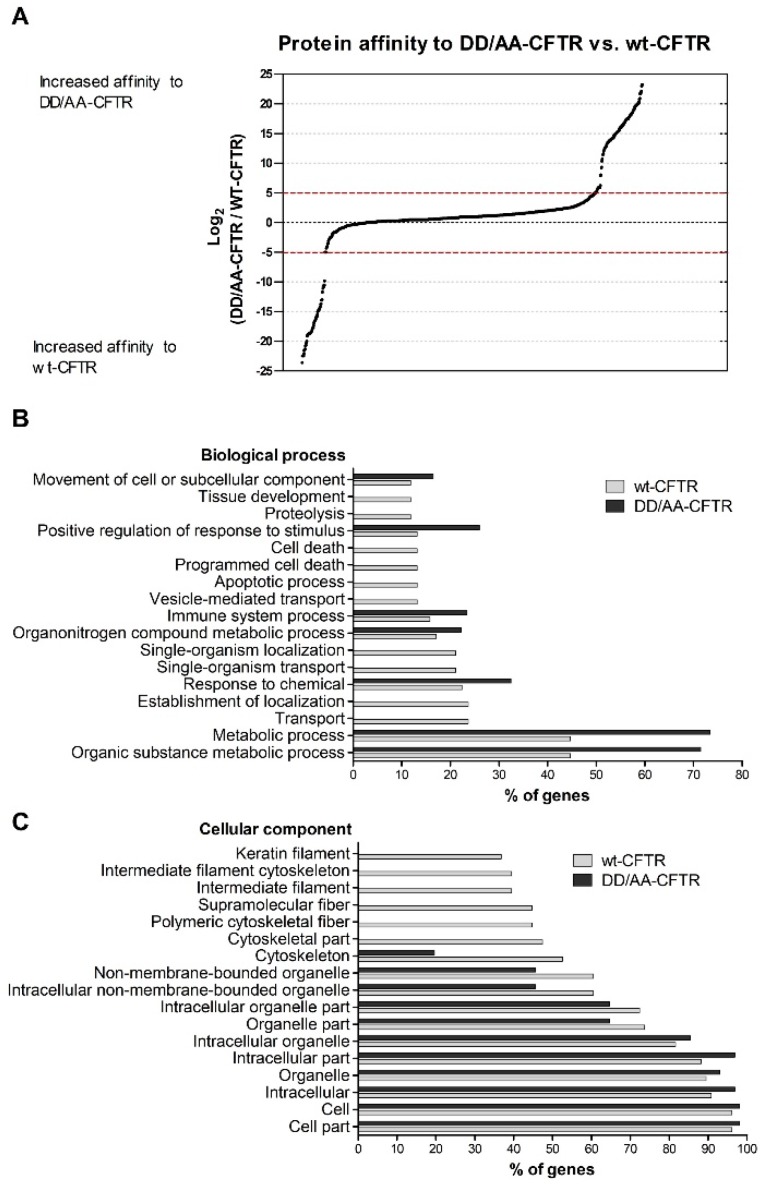
Differential protein interactions detected for DD/AA-CFTR versus wt-CFTR. (**A**) Log_2_ plot of the protein abundance ratio for DD/AA-CFTR versus wt-CFTR. Proteins were identified by LC-MS/MS, and black dashed lines indicate the threshold (log_2_ = ±5). Experiments were performed in triplicate (N = 3). GO analysis for differential interactors of DD/AA- versus wt-CFTR. (**B**) Biological process and (**C**) cellular component represented for DD/AA-CFTR and wt-CFTR using Database for Annotation, Visualization and Integrated Discovery (DAVID). From the total of 1150 proteins, the 161 with more affinity to DD/AA-CFTR and the 77 with more affinity to wt-CFTR were used to find the GO term biological process enriched in both subsets. *p*-value < 0.05.

We have used iBAQ values to calculate the differential affinity of DD/AA- versus wt-CFTR, as described above (Materials and Methods). The differential affinity of each immunoprecipitated protein between DD/AA-CFTR and wt-CFTR was calculated as the base 2-logarithm of the ratio of the median for each protein for the DD/AA-CFTR IP with the median expression values. We assumed all the proteins outside the threshold of log_2_ ratio ±5 were differential interactors between DD/AA-CFTR and wt-CFTR. Among the 1150 proteins identified, we obtained 161 proteins with higher affinity for DD/AA-CFTR (above the threshold log_2_ ratio of five) and 77 proteins with higher affinity for wt-CFTR (below the threshold log_2_ ratio of −5) (Datasets S2A,B, respectively, and Figure 1A).

Using DAVID (GO Version 6.8), we analyzed the proteins with higher affinity to DD/AA-CFTR and those with higher affinity to wt-CFTR (based on the threshold previously established) to find the respective GO terms. For GO term BP (biological process), we found an enrichment in vesicle-mediated transport for wt-CFTR when compared with DD/AA-CFTR (Figure 1B). As inactivation of the diacidic motif prevents CFTR exit from the ER, this is likely to reduce its association with the machinery involved in protein transport. For the GO term CC (cellular component), we observed an enrichment in cytoskeleton components for wt- when compared to DD/AA-CFTR (Figure 1C).

### 3.2. Recognition of CFTR AFT Retention Motifs

To further characterize the ERQC, we identified proteins involved in recognizing the AFTs (i.e., at the third ERQC checkpoint). Previously, we used differential interactomics to identify such regulators [18]. Now, we used a pull-down approach in non-CFTR expressing CFBE cells with peptides mimicking such CFTR regions: 10 amino acid-long peptides corresponding to the CFTR sequence around each of the AFTs (see Materials and Methods). The pull-down was performed using whole cell lysates in order to obtain a larger number of proteins and avoid any “filtering” through this step.

**Figure 2:**
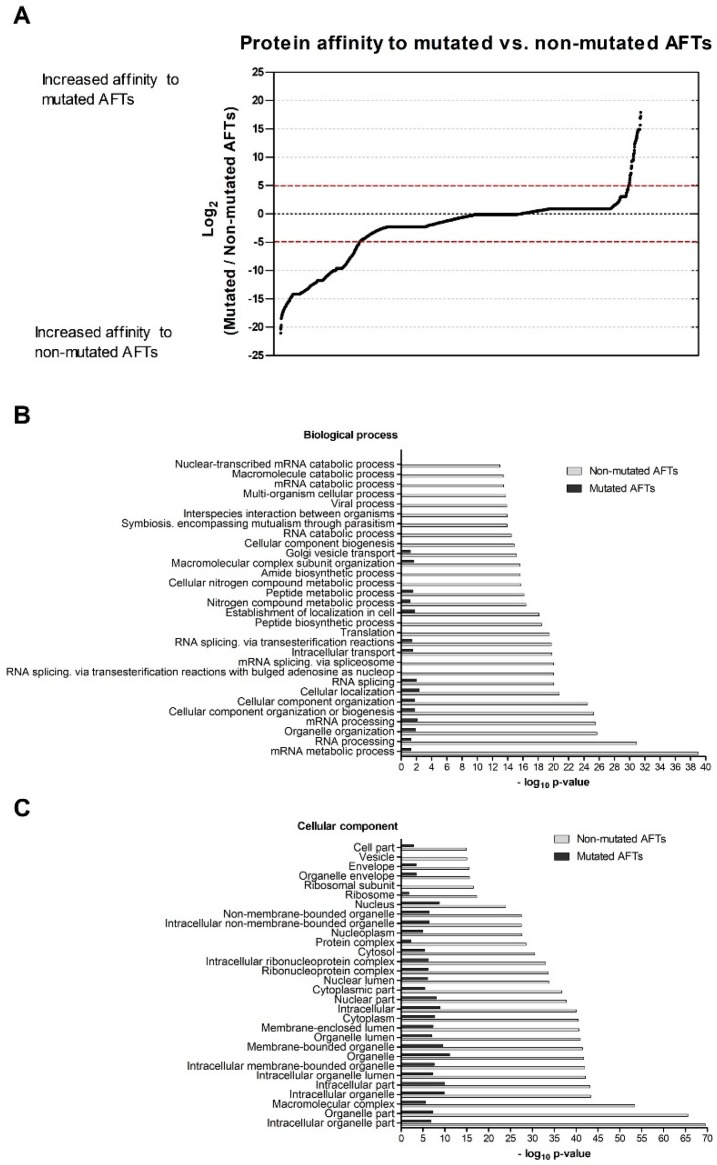
Differential protein interactions detected for peptides mimicking wt- and mutated AFT motifs. (**A**) Pull-down of proteins from cystic fibrosis bronchial epithelial (CFBE) parental cells that bind to non-conjugated beads (control) and beads conjugated to peptides corresponding to mutated and non-mutated AFTs. In total, 3393 proteins were identified by the LC-MS/MS method. AFT interactors were quantified using intensity-based absolute quantification (iBAQ) values and compared using the ratio log_2_(mutated AFT interactors/non-mutated AFT interactors); −5 ˂ log_2_ ˂ 5 were considered non-specific proteins. Experiments were performed in triplicate (N = 3). (**B**) Biological process and (**C**) cellular component of proteins with increased affinity to mutated vs. non-mutated AFTs. DAVID was used to evaluate the enriched GO terms in the 114 proteins with increased affinity to mutated vs. 742 proteins with increased affinity to non-mutated AFTs. −log_10_(*p*-value) was considered to evaluate the statistical significance for all categories.

LC-MS/MS results of pulled-down extracts identified a total of 3393 proteins (Dataset S3, Figure 2A). Using a similar cutoff as above, 742 proteins were identified with an increased association with non-mutated AFTs and 114 with an increased association with mutated AFTs (Datasets S4A,B, respectively). The number of proteins strongly interacting with non-mutated AFTs was higher, as well as the interaction fold range. The mutated peptides correspond to non-physiological sequences and, thus, are used mainly to discriminate which interactions are AFT-motif-specific.

The list of AFT interactors was analyzed using DAVID to evaluate the most represented protein families (Figure 2). For GO term BP, mRNA metabolic process, RNA processing, and organelle organization were the most significantly-enriched categories in the interactors of PeptR (non-mutated AFTs) (Figure 2B). For GO terms CC, intracellular organelle part, organelle part, and macromolecular complex were the most enriched (Figure 2C). The number of proteins with increased affinity to the PeptK (mutated AFTs) interactors was low, showing no significant enrichment, which was expected considering that the sequences are “non-physiological”.

### 3.3. Comparative Analysis of Interactors of CFTR Variants

To extract more information from the large datasets of interacting proteins identified, we then performed an extended comparison of the four different datasets obtained here (Datasets S1, S2, S3, S4) with the interactomes of F508del-CFTR and F508del-4RK-CFTR that we previously published [18].

**Figure 3:**
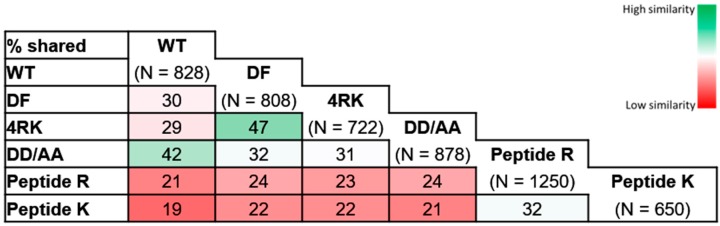
Similarity index for protein interactors. Heat map of the Jaccard similarity indices for the indicated pairwise comparisons. Number of genes in the interactome list indicated in the horizontal row in common with the interactome shown at the top (WT: wt-CFTR interactome, DF: F508del-CFTR interactome, 4RK: F508del-4RK-CFTR interactome, DD/AA: DD/AA-CFTR interactome for full-length CFTR and Peptide R and K interactomes). % shared (similarity) was obtained using the Jaccard similarity index.

We started by a global comparative analysis to identify common proteins among all datasets (WT: wt-CFTR interactome, DF: F508del-CFTR interactome, 4RK: F508del-4RK-CFTR interactome, DD/AA: DD/AA-CFTR interactome, PeptR, pull-down of non-mutated AFT motifs, PeptK, pull-down of mutated AFT motifs) and calculated for each pair the Jaccard similarity index (Figure 3, Dataset S5). Interestingly, greater similarity seemed to occur within the four full-length CFTR variants (range 30–47%) and within the two peptide pull-down datasets (32%); this suggests that proteins interacting with CFTR/peptide are very much dependent on the presence of full-length protein and that the presence of the isolated AFT motifs modifies greatly the interactome. A second relevant observation is that within the four datasets for full-length CFTR variants, greater similarity occurred for the pairs WT/DD/AA (42%) and F508del/F508del-4RK (47%), suggesting that the folding status is a stronger determinant for the interactome when compared to the trafficking status.

**Figure 4:**
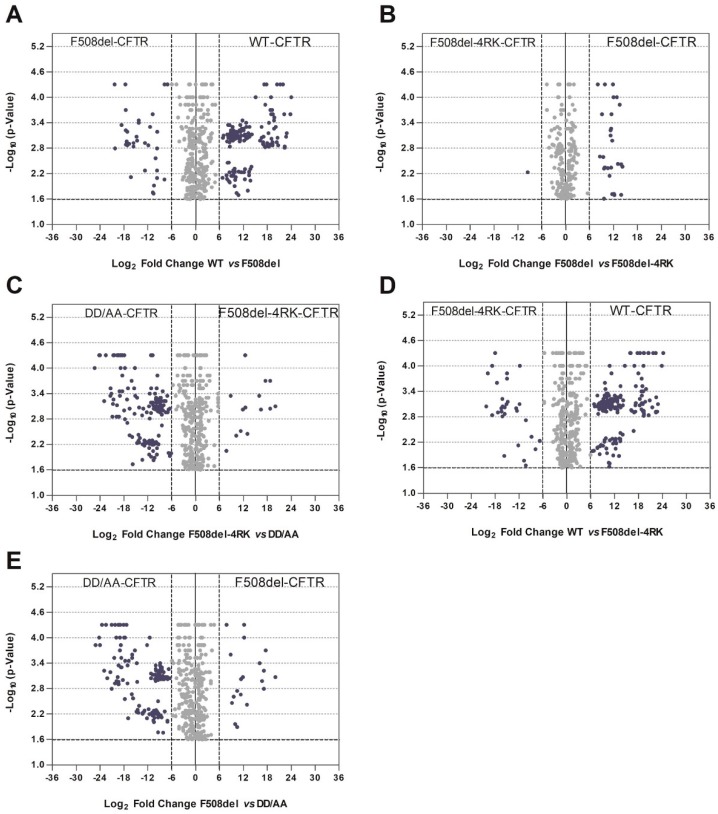
Volcano plots for pairwise comparisons of full-length CFTR variants. Significance is represented by −log_10_ *p*-value calculated using permutation tests (10,000 permutations), and the protein affinity for the two variants under analysis is assessed as described in the text. Blue dots represent hits selected based on *p*-values < 0.025 and |fold change| >6. **A**–**E**: Pairwise comparisons as indicated in the figure.

We then performed pairwise comparisons within the four variants (Figure 4 showing comparisons of WT/F508del, F508del/F508del-4RK, DD/AA/F508del, F508del-4RK/WT, DD/AA/F508del-4RK, plus the comparison WT/DD/AA shown in Figure 1, Dataset S6) and bioinformatics analysis for the proteins with statistically-significant stronger affinity in each pairwise comparison. For these comparisons, we used a holistic approach and higher stringency to reduce the number of proteins detected (see Materials and Methods). Significant proteins were identified using the thresholds: −log_10_(*p*-value) ≥ 1.6 (corresponding to *p* < 0.025) (log_2_(Condition 1) − log_2_(Condition 2)|) ≥ 6.

Comparison of the wt- and F508del-CFTR interactomes (Figure 4A and Table S1A–D) recapitulated previous observations reporting an enrichment in GO terms related to membrane localization, ER targeting (BP), and for cytoskeleton-related categories (CC) for wt-CFTR [15]. For F508del-CFTR, there was an enrichment in mRNA metabolism and ribosomal association (BP) or intracellular compartments (CC).

The pairwise comparison of F508del-CFTR to F508del-4RK-CFTR (Figure 4B), although previously published by us [18], was repeated here with more stringent thresholds. Results recapitulated the previous ones, again showing an enrichment in RNA and intracellular organelles’ related terms for F508del-CFTR.

Interestingly, comparison of F508del-4RK-CFTR and DD/AA-CFTR (Figure 4C and Table S1E–H) revealed an enrichment in categories for DD/AA-CFTR very similar to those detected for wt-CFTR (protein targeting to ER, targeting to membrane) and in categories for F508del-4RK-CFTR very similar to those observed for F508del-CFTR (RNA metabolism terms, intracellular organelles).

Comparison of F508del-4RK-CFTR with wt-CFTR (two variants that reach the plasma membrane) is shown in Figure 4D and analysis of the GO terms enriched in differential interactors in Table S1K–L. Among wt-CFTR interactors, there was an enrichment in protein targeting to the ER and to membrane-related terms (BP), whereas for F508del-4RK-CFTR interactors, RNA-related terms and intracellular organelles were enriched (similar to what was described above for F508del-CFTR interactors).

Finally, we compared the two variants that were retained (F508del- and DD/AA-CFTR) (Figure 4E). Enriched terms for F508del-CFTR were once again mainly related to RNA or regulation of gene expression (BP) and intracellular organelle associated (CC), whereas for DD/AA-CFTR categories related to intracellular transport, targeting to the ER or trafficking vesicles was detected.

Overall, the pairwise analyses shown in Figure 4 and Table S1 confirmed what was suggested by the Jaccard similarity index (Figure 3), that the interactomes (and the categories enriched) were closer for variants with higher folding affinities (wt and DD/AA that were properly folded versus F508del and F508del-4RK that had deficient folding) than for those with trafficking similarities (DD/AA and F508del that were retained versus wt and F508del-4RK membrane-located variants).

A qualitative analysis covering only proteins present in at least two replicates (Figure S2) evidenced more striking differences at the level of protein folding machinery-related terms. For this analysis, the protein lists for F508del-, DD/AA-, F508del-4RK-, wt-CFTR (Dataset S1 and [18]), and their pairwise combinations were assessed for enriched GO terms related to ER retention and folding processes. We found that the protein folding term was enriched in folding mutants (F508del- and F508del-4RK-CFTR) in comparison to wt- and DD/AA-CFTR (Figure S2). Interestingly, when the wt- and DD/AA-CFTR interactors were listed together with those of F508del- and F508del-4RK-CFTR, a reduction in this enrichment was observed (Figure S2). Finally, PM region was found to be enriched in wt- and F5808del-4RK-CFTR, but not in F508del- or DD/AA-CFTR (Figure S2).

We also identified proteins that were only present in the F508del-CFTR interactome, but neither in the DD/AA-CFTR, nor in the wt-CFTR interactomes. As above, for this analysis, only the proteins present in at least two biological replicates were considered. Using these criteria, the Venn diagram in Figure S3A shows the comparison of proteins identified between replicates of F508del-CFTR, DD/AA-CFTR (represented as DD/AA), and wt-CFTR (represented as WT). From a total of 808 proteins identified in the F508del-CFTR interactome, 243 proteins were not present in neither the DD/AA-, nor the wt-CFTR interactomes, suggesting that these are proteins specifically involved in F508del-CFTR folding and ER retention-related processes (Figure S3A, Dataset S7). Comparison of these data with a proteome map study for ER membrane proteins [33] revealed that F508del-CFTR, contrary to wt- and DD/AA-CFTR, interacted exclusively with 24 ER membrane proteins (Figure S3B, Table S2).

Finally, the proteins exclusively interacting with either F508del-, wt-, or DD/AA-CFTR (Figure S3A) were analyzed for enriched GO terms (Figure S4). RNA processing and protein folding were the BP more enriched for F508del-CFTR. Among these proteins, we found membrane-enclosed as a CC term enriched in F508del-CFTR and DD/AA-CFTR. In wt- and F508del-CFTR, cytoskeleton was also found to be enriched.

### 3.4. Comparative Analysis of Interactors Involved in AFT Recognition

We applied a similar analysis to the one described in the previous section to the proteins interacting with the AFT and control peptide sets. Besides the comparison between interactors of the two sets of peptides (Figure 2), we performed comparisons of these interactomes with those of wt-, F508del-, and F508del-4RK-CFTR.

**Figure 5:**
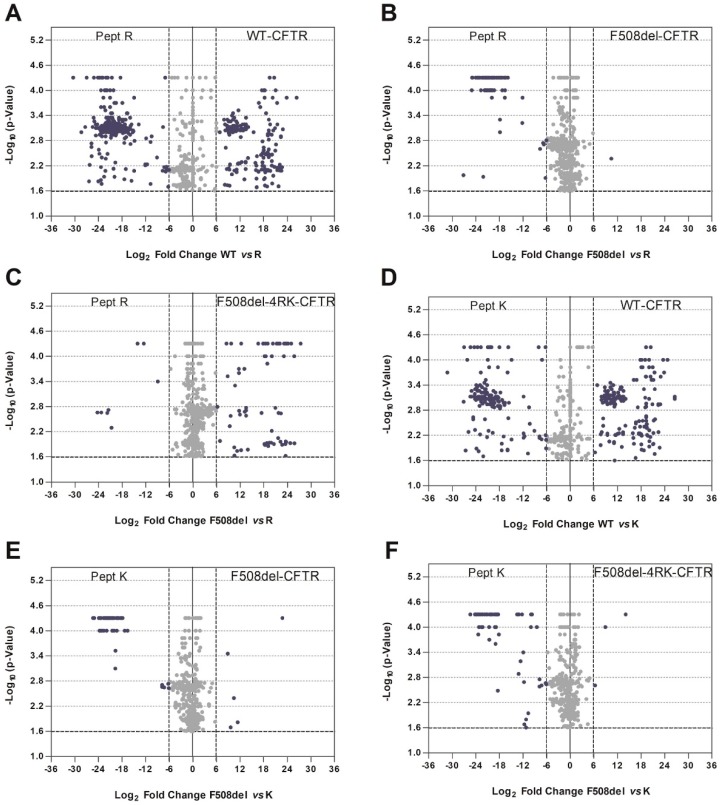
Volcano plots for pairwise comparisons of the interactomes related to AFT recognition. Significance is represented by−log_10_ *p*-value calculated using permutation tests (10,000 permutations), and the protein affinity for the two variants under analysis is assessed as described in the text. Blue dots represent hits selected based on *p*-values < 0.025 and |fold change| >6. **A**–**F**: Pairwise comparisons as included in the figure.

Comparison of the peptide interactomes with that of wt-CFTR (Figure 5A,C and Table S3A–H, Dataset S5) showed an enrichment in protein targeting categories for wt-CFTR and an enrichment in mRNA and protein biosynthesis/translation categories for the peptides interactomes (and some fewer specific categories for PeptK, which correspond to sequences that are “non-physiological”).

Interestingly, when we compared the F508del-CFTR interactome with those of the peptides (Figure 5B,D and Table S3I–N), the number of differential interactors was very small, suggesting some similarity in these datasets, which was expected to include proteins scrutinizing CFTR (or other secretory pathway proteins) in the ER. Examples of these common proteins detected include calnexin, components of the COPII machinery or even the translocon subunit Sec62.

This analysis providing comparison of interactomes from peptides and full-length CFTR, together with the results presented above (Figure 2), evidences the specificity of the AFT recognition in terms of function (protein retention) and localization of activity (the ER).

### 3.5. Integrated Analysis of ERQC Interactomes

After the pairwise comparisons for the relevant interactomes, we performed an integrated analysis of the full lists of interacting proteins using principal component analysis (PCA).

**Figure 6:**
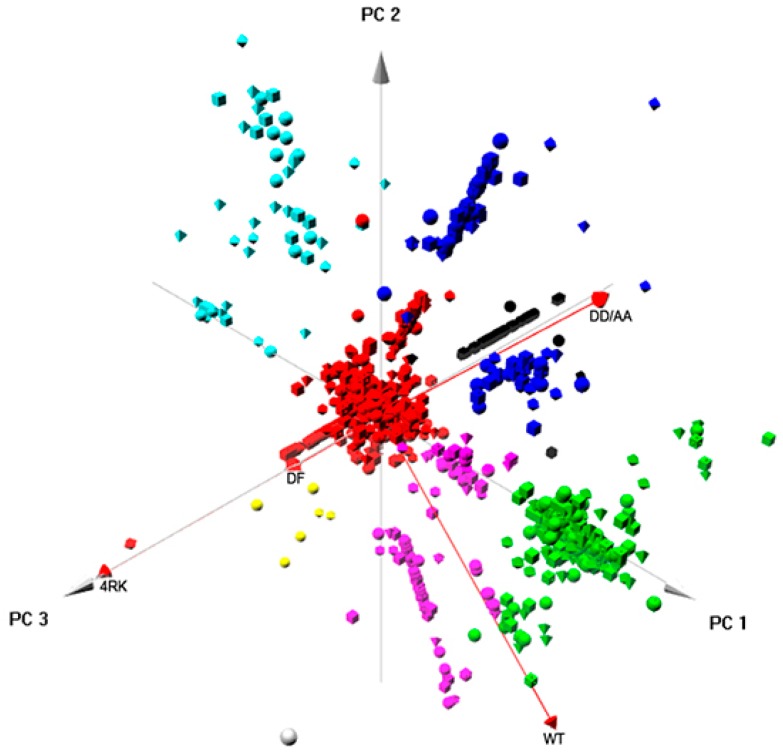
Component analysis for full-length CFTR variants. PCA was performed using the package “pca3d” from R programming [31]. Data corresponding to the combinations of the median of the amount of each protein immunoprecipitated with each CFTR variant calculated as described above were used for PCA analysis. The PCA scores corresponding to the three main components are plotted in a scatter plot in which the position along the axes show the PCA score of the protein. Clustering was performed using hierarchical agglomerative clustering through the standard R function “hclust” and using average linkage as a clustering method.

We first analyzed the set of proteins interacting with the four full-length CFTR variants (WT, F508del, F508del-4RK, and DD/AA) using as above the subtraction of the log_2_ of the medians of the amount of a protein isolated in two different variants. The results are shown in Figure 6; in this analysis, the first two components explain 96% of the differences in the samples; the third adds a further 3%; and the fourth the remaining 1%. Clustering was performed with HAC using hclust heuristics. The total number of proteins clustered into eight different groups (see Materials and Methods), and the red arrows represent the four CFTR variants under consideration. Results showed that F508del, F508del-4RK, and DD/AA aligned in the same axis with the same direction. DD/AA and WT aligned through PC1 axis although with a different PC2 direction.

We then performed a GO enrichment analysis to identify the terms in each cluster (Table S3). The large central cluster (red) with over 3000 proteins is most likely a cluster of non-distinctive genes, where no observable differences among CFTR variants has been discerned. The black cluster (43 proteins) shows some dominance of proteins annotated with RNA metabolism terms and processing (BP) and nuclear and intracellular organelle-related terms (CC). The green cluster (268 genes) shows dominance of proteins annotated to ER targeting and localization (BP) and ribosome/cytosol-related terms (CC). The blue cluster (107 proteins) shows some dominance of proteins annotated with translation, regulation of translation, translation initiation (BP), and vesicle-related ones (CC). The cyan cluster (58 proteins) is enriched in proteins annotated in terms similar to the ones in the black cluster (RNA processing, regulation of RNA stability for BP, nuclear and intracellular organelle for CC). The purple cluster (122 proteins) groups proteins with enrichment for terms related to vesicle-mediated transport or membrane invagination (BP) and cytoskeleton or filaments (CC). Finally, a small group (yellow cluster with only six proteins) was detected for which the only enrichment detected was for terms related to transport vesicles (CC). Overall, this analysis identified five main categories that can be distinguished: RNA processing; ER targeting and localization; translation; trafficking; transport vesicles; and among these clusters, protein–protein interaction networks could also be identified (Figure S5).

**Figure 7:**
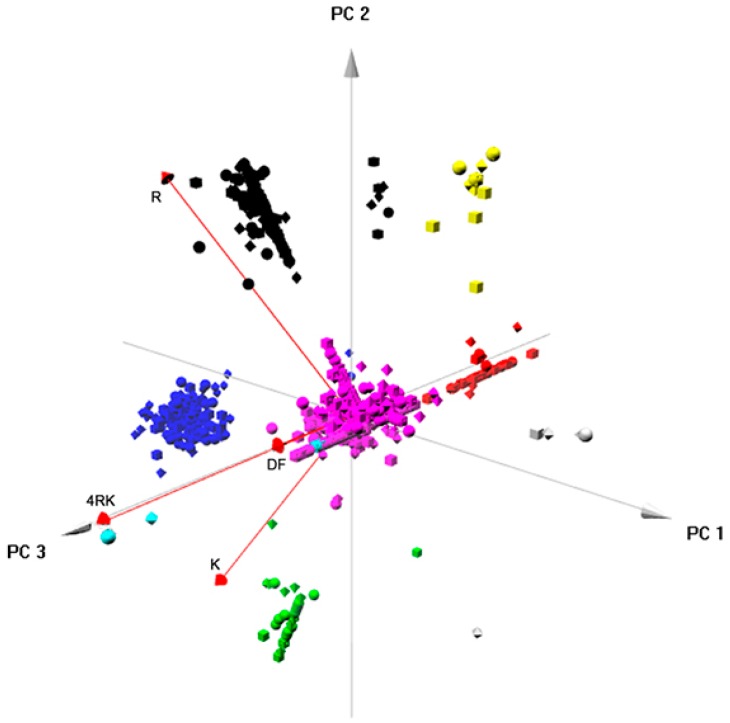
Component analysis for the protein sets corresponding to the arginine-framed tripeptide-related checkpoint. PCA was performed using the package “pca3d” from R programming [31]. Data corresponding to the combinations of the median of the amount of each protein immunoprecipitated with each CFTR variant (or pulled-down with each set of peptides) calculated before were used for PCA analysis. The PCA scores corresponding to the three main components were plotted in a scatter plot in which the position along the axes shows the PCA score of the protein. Clustering was performed using hierarchical agglomerative clustering through the standard R function “hclust” and using average linkage as a clustering method.

We performed a similar analysis comparing the protein lists related to regulation of CFTR ER exit by the AFTs (full-length F508del- and F508del-4RK-CFTR plus PeptR and PeptK). The results (Figure 7) showed that, as above, the first two components explained 96% of the differences in the samples; the third added a further 3%; and the fourth the remaining 1%. The total number of proteins clustered into eight different groups for which bioinformatics analysis was then performed (Table S4). As above, the large central cluster (purple) was most likely a cluster of non-distinctive genes, where no observable differences among variants has been discerned. The black cluster (180 genes) showed dominance of proteins annotated to metabolic processes (BP). The red cluster (48 proteins) was enriched in proteins related to RNA processing and translation initiation (BP). The green cluster (34 proteins) grouped proteins enriched with terms actin filament or organelle/complex assembly/organization (BP). The blue cluster (178 proteins) included proteins with terms related to RNA splicing, but also cytoskeleton (BP). The cyan cluster, although quite small (11 proteins), was enriched in terms related to protein folding and modification (BP). The yellow cluster (15 proteins) was enriched in cytoskeleton- and microtubule-related terms (BP). Finally, the grey cluster (only five proteins) was enriched in terms related to ion or mitochondrial transport (BP). Overall, this analysis identified seven main categories: metabolic processes; RNA processing and translation; complex assembly and organization; RNA splicing; protein folding; cytoskeleton and microtubules; and ion/mitochondrial transport. As above, and among these clusters, we were able to identify protein–protein interaction networks (Figure S6). Compared to the PCA described above, in this case, protein folding was evidenced as a distinctive category, which was absent when the full-length variant interactomes were compared.

## 4. Discussion

CFTR folding and exit from the ER is controlled by the ERQC machinery, which scrutinizes the folding status and trafficking competence of the protein. Such assessment involves recognition of specific motifs in the CFTR sequence, such as the AFTs for ER retention and the diacidic code implicated in ER exit [5,12]. Whereas the AFT motif functions as a negative signal responsible for preventing the exit of unfolded proteins, the diacidic one is a positive one mediating association with the COPII machinery directly involved in ER export.

When CFTR achieves its native structure, the AFTs are buried within the protein structure, and it is able to interact with the COPII machinery through exposure of the diacidic exit code being exported from the ER to the Golgi apparatus. However, misfolded F508del-CFTR, which aberrantly exposes these ATFs, is retained in the ER, being trapped by the ERQC machinery and targeted to degradation [5,12].

Analysis of CFTR interactomes has provided information on relevant protein–protein interactions and how these can be modulated along with rescue of the most common mutant protein [15], or more recently how such interactions may differ between mutations causing distinct cellular defects [34], or may reflect the response to correctors or potentiators. Here, and following previous studies by our group and others, clarifying the mechanisms that distinguish between folded and misfolded CFTR retaining and targeting the latter for degradation, we aimed here at using interactomics of CFTR variants characteristic of the ERQC checkpoints and performed a global comparative analysis of the identified protein interactors to shed light on the trafficking steps involved.

We first focused on how the disruption of the diacidic exit code impacts the CFTR interactome. Abrogation of the exit code decreased association with proteins involved in vesicle-mediated transport or related to the cytoskeleton (Figure 1C). Interestingly, when performing this comparison, we observed an enrichment in terms related to cell death for wt-CFTR. Although still a controversial subject, this is in agreement with reports identifying CFTR as a pro-apoptotic factor [35], which may depend on CFTR ability to transport glutathione [36] and involve activation of TMEM16F [37]. Among the proteins with stronger interactions with DD/AA-CFTR, we found examples of trafficking pathway-related proteins such as GET4, a protein involved in the alternative ER membrane insertion of membrane proteins [38] and reported previously as participating, together with BAG6 and UBL4, in promoting efficient degradation of misfolded ER proteins [39], or TRIP10 (CIP4), a protein that has been associated with EMT, which is also characteristic of CF [40] and which participates in vesicle traffic localizing to Rab5-positive endosomes [41], promoting, e.g., the trafficking of epithelial growth factor receptor (EGFR) from the Rab5-positive endosomes to lysosomes. This list of interactors suggests that these proteins strongly associating with CFTR with an abrogated diacidic exit code may have either a direct role (a new role of TRIP10 in regulating the ERQC) or its effect may appear from a possible feedback from late trafficking events (such as the ones involving Rab5 in which TRIP10 has been implicated and considering that Rab5 was reported to facilitate CFTR trafficking from the PM to early endosomes [42]).

We then addressed how proteins interacting with the AFT motifs may regulate CFTR exit from the ER. Among these lists, we detected several proteins previously identified as possible regulators of F508del-CFTR including UBE2I, UBA52, UBA2, or CHD4 [43]. Among the ones with stronger interactions to non-mutated AFTs, there seems to be large representation of relevant general folding/ proteostasis components such as GEFs for trafficking GTPases or folding sensors. These results suggest that recognition of AFTs is a general mechanism for folding assessment relying on ubiquitous (and pivotal) components of the cellular machinery. We have shown previously that knocking down of relevant folding components such as Hsp70 does not promote folding of F508del-CFTR [7], and others also have shown that proteasome inhibition does not rescue F508del-CFTR per se [44]. Taking this into consideration, it is likely that these AFT interactors are not the ideal ones to modulate in order to rescue F508del-CFTR. Previously, our comparison of the interactomes of full-length F508del- and F508del-4RK-CFTR identified regulators (such as KIFC1 or YWHAE) that when knocked down rescued F508del-CFTR to the cell surface [18]. Therefore, the AFT-interacting retention proteins are likely part of larger protein complexes (including other proteins such as KIFC1) that bind the misfolded protein targeting it to degradation [18]; these rather than the AFT interactors are more likely to be amenable to modulation to promote rescue.

After looking more in detail at proteins putatively involved at the different checkpoints, we performed an extended comparative analysis of the interactor lists obtained here. A first comparative analysis (Figure 3) identified a greater similarity between CFTR variants with similar folding patterns; increased Jaccard indexes were observed for the pairs wt-/DD/AA-CFTR (42%) and F508del-/F508del-4RK (47%), the first two of which achieved correct folding and the latter two with folding defects. Lower similarities were observed when we compared the interactomes of variants that share the same subcellular localization: DD/AA- and F508del-CFTR were both at the ER, but the Jaccard index was only 32%; while wt- and F508del-4RK-CFTR both reached the PM, but the index was only 29%. These numbers are in fact similar to the Jaccard index for the wt- and F508del-CFTR (30%), evidencing that variants that differ in their folding status are more dissimilar in terms of interactors, disregarding their localization.

**Figure 8:**
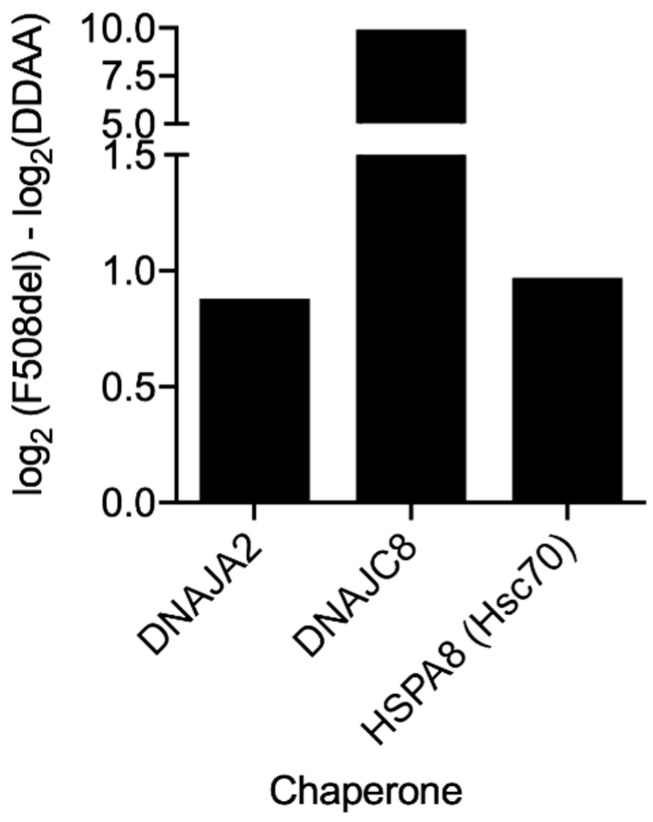
Association of f508del-CFTR with chaperones compared to DD/AA-CFTR. The protein affinity for the two variants under analysis was assessed by calculating log_2_(Condition 1) − log_2_(Condition 2)| and shown for variants with −log_10_(*p*-value) ≥ 1.6 (corresponding to *p* < 0.025) as in Figure 4 (see Materials and Methods).

Such observations were confirmed when we performed pairwise comparisons and GO analysis, using the DAVID database, on the differential interactors (Figure 4 and Table S1). Interactors of correctly-folded variants were enriched in terms related to membrane localization and ER targeting, whereas misfolded variants interacted with proteins related to mRNA metabolism and ribosomal association or with intracellular compartments. It is particularly striking that the enriched GO terms for wt- vs F508del-4RK-CFTR were very similar to those in the comparison wt- vs. F508del-CFTR. Equivalent behavior was observed if we look at enriched terms for DD/AA- vs. F508del-CFTR or DD/AA- vs. F508del-4RK-CFTR. Examples for such differential interactors include proteins such the molecular chaperones HspB1 [45,46] and ERP44 (endoplasmic reticulum resident protein 44), both of which were in increased association with F508del- and F508del-4RK-CFTR over either wt- or DD/AA-CFTR. Detailed analysis (highlighted in Figure 8) also showed an increased interaction of F508del- over DD/AA-CFTR with chaperones, namely the heat shock cognate 71KDa protein (HSPA8/Hsp73) or members of the Hsp40/DNAJ family such as DNAJA2 or DNAJC8 [47]. This result once again suggests that although both being retained, F508del- and DD/AA-CFTR have distinct interactomes, and these differences include members of major chaperone families.

Comparison of our datasets with one of the most comprehensive CFTR interactomes [15] showed major overlap, that is about 34%, 42%, and 76% for the pairs F508del/F508del-4RK, WT/DD/AA, and PeptR/PeptK, respectively, a relevant figure considering the methodological differences.

On extending our recent characterization of the interactome of F508del-4RK-CFTR, to further dissect the ERQC, we observed that misfolded F508del-CFTR and F508del-4RK-CFTR established less interactions when compared to wt-CFTR and DD/AA-CFTR interactomes (Figure 3). Interestingly, we observed that F508del-CFTR (n = 808) established more interactions when compared to F508del-4RK-CFTR (n = 722) and that DD/AA-CFTR (n = 878) also presented more interactions than wt-CFTR (n = 828). Pairwise comparisons for variants with similar folding status suggested that the number of interactions was higher for the variants that were retained. GO enrichment analysis was performed for the pairwise groupings and revealed a dominance for proteins annotated with protein folding among the interactors of F508del- and F508del-4RK-CFTR when compared to wt- and DD/AA-CFTR (Figure S2). When protein interactors of wt- or DD/AA-CFTR were analyzed together with the F508del- and F508del-4RK-CFTR interactomes (full set of proteins grouped), such enrichment decreased (Figure S2). These results confirm the established knowledge that misfolded CFTR is recognized as aberrant by the ERQC system, being retained in the ER and consequently having more prolonged contact with the folding machinery than wt-CFTR [3]. Interestingly, such enrichment was observed also for F508del-4RK-CFTR, which traffics to the PM despite its folding defect. That is probably the reason for which we detected ubiquitin ligases such as RFN14 or proteasomal subunits such as PSMA7 with increased association with F508del-4RK-CFTR; such observations are in agreement with the peripheral quality control that assesses CFTR folding status after exiting the ER [48,49] and also with refined characterization of other ubiquitin ligases as potential targets to rescue F508del-CFTR [43,50]. The detection of such components of the peripheral quality control may also account for the reduced amount of F508del-CFTR that may reach the cell surface, being thus the substrate for this machinery.

By comparing the wt-CFTR, F508del-CFTR, and DD/AA-CFTR interactomes, we identified 243 proteins that are only present in the F508del-CFTR interactome, but neither in the DD/AA-CFTR, nor in the wt-CFTR interactome (Figure S3A, Dataset S7). Interestingly, 24 proteins among these 243 proteins belong to the ER membrane class, as identified by a proteome map study [33] (Figure S3B and Table S2). GO analysis identified RNA-related processes and protein folding as the BP enriched among these proteins (Figure S4). Previously, also an enhanced recruitment of proteins involved in RNA processing in F508del-CFTR interactome was reported [15]. Thus, this result suggests that most of the protein associations are related to the folding defect characteristic of mutated CFTR and the associated ER retention. When these 243 unique proteins were crossed with the F508del-4RK interactors, we detected 201 common proteins, once again confirming the similarity of these two variants (F508del/F508del-4RK interactors not detected for WT or DD/AA).

Finally, integrated approaches using PCA provided relevant insight into the global pattern of protein interactions involved in the ERQC for CFTR. When analyzing globally the interactomes of the four full-length CFTR variants (Figure 6), clustering enabled the identification of five major classes of proteins (that corresponded also to interaction networks; Figure S5) related to: RNA processing; ER targeting and localization; translation; trafficking/endocytosis; vesicles, once again, through vectorial representation, puttingF508del- and F508del-4RK-CFTR closer, distant from WT- and DD/AA-CFTR. Global analysis of the four interactomes related to AFT-mediated trafficking (Figure 7) identified six clusters/protein interaction networks (Figure S6) related to: the metabolic process; RNA processing/translation; complex assembly and organization; RNA splicing; folding; cytoskeleton/microtubule. Interestingly, while some overlap was present, the two global analyses highlighted different processes: they confirmed the distance between variants and shed light onto the overall functioning of these checkpoints. Whereas comparison of full-length variants resulted in more general classes of proteins, comparison of AFT-related interactomes was more focused on events that relate more to CFTR biosynthesis and ER membrane insertion.

The global analysis performed provides an overview of the proteins that regulate CFTR folding, trafficking, and degradation; we detected great similarities in terms of overlap, similar enrichments, and relevant common proteins for CFTR variants that share the same folding status when compared to those with similar locations. Overall, the insight brought by the current study is the first to show such similarities for the same time, applying it not to disease-causing mutations, but to variants that allow the characterization of the ERQC. In addition to shedding light onto the basic biology of CFTR, it can also and ultimately contribute to further reveal pathways that can be explored in terms of therapeutic modulation for the benefit of CF patients.

## Supplementary Materials

The supplementary materials are available online at https://www.mdpi.com/2073-4409/8/4/353/s1.
