# Supplementary material for: Folding Status Is Determinant over Traffic-Competence in Defining CFTR Interactors in the Endoplasmic Reticulum
Source: Cells. 2019 Apr 14;8(4):353. doi: 10.3390/cells8040353 (PMC6523853; doi:10.3390/cells8040353)
Supplement: Supplementary file 1 [file cells-08-00353-s001.zip › Supplementary materials/Supplementary Materials.docx]

**Supplementary Materials**

**Figures**

**Fig.S1 - CFTR expression in CFBE cells stably expressing DD/AA-CFTR**. CFBE cells expressing DD/AA-CFTR (lane 1-6), wt-CFTR (lane 7) and F508del-CFTR (lane 8) were grown at 37ºC or 26ºC and in some cases incubated with VX-809 (3µM) for 24h or DMSO (vehicle compound). NT, non-treated. **A)** Western blot (top) showing both mature form band C (~170kDa) and immature form band B (~140kDa) of CFTR protein. Calnexin (90kDa) was used as a loading control (bottom). **B)** Amount of immature form of CFTR in cells expressing DD/AA-CFTR, normalized to the loading control and wt-CFTR. Data plotted as the mean ± SEM, n=4, * p<0.05. Statistical analysis was performed using two-tailed unpaired students t-test. **(C)** Expression of the CFTR traffic reporter in the wt-, F508del- and DD/AA-CFTR variants. Representative widefield fluorescence microscopy images of CFBE cells expressing the several variants of the CFTR traffic reporter. The three pannels for each variant show hoechst-stained nuclei, the mCherry fluorescence originating from all CFTR molecules, and the immunofluorescence of PM-located CFTR in unpermeabilized cells (Flag tag). No siRNA was added to cells. Scale bar: 50 µm. **(D)** Quantification of the mCherry fluorescence for the experiment described in the pannel A. The box plots show the integrated fluorescence signal for single cells. The number of measured cells is shown between brackets. Stars: p < 0.05, Welch two sample t-test.

**Fig.S2 – Bioinformatics analysis for proteins present in at least two replicates.** **Functional enrichment analysis of the genes found in total WT, DF, 4RK and DD/AA group in the current study.** The genes for wt-CFTR, for F508del-CFTR, for F508del-4RK-CFTR, for DD/AA-CFTR and their combinations were used to find the GO terms – Biological process (BP) / cellular compartment (CC) enriched in all subsets. p-value<0.05.

**Figure S3 - Analysis of F508del-, DD/AA- and wt-CFTR groups.** Venn diagram **A)** showing the proteins overlap in F508del-, DD/AA- and wt-CFTR groups and **B)** ER membrane proteins identified in these groups as observed in the ER membrane proteome map study Hung et al., 2017.

**Figure S4 - Functional enrichment analysis of the genes found just in DF, WT and DD/AA group in the current study.** The 243 genes for F508del-CFTR, 77 for wt-CFTR and 66 for DD/AA-CFTR were used to find the GO terms – Biological process (BP) / cellular compartment (CC) enriched in all subsets. p-value<0.05.

**Figure S5 – Protein interaction networks for clusters detected in PCA for CFTR variants.** Protein-protein interactions were analyzed using APID and Cytoscape was used for visualization of the networks.A – black cluster; B – green cluster; C – blue cluster; D – cyan cluster; E – purple cluster.

**Figure S6 – Protein interaction networks for clusters detected in PCA for peptide interactors.** Protein-protein interactions were analyzed using APID and Cytoscape was used for visualization of the networks. A – black cluster; B – red cluster; C – green cluster; D – blue cluster; E – cyan cluster; F- yellow cluster; G – grey cluster.

**Tables**

**Table S1 -** **GO analysis for differential interactors of full-length CFTR variants.** Biological process and cellular component are represented for each pair under comparison.

**Table S2** – List of 24 ER membrane proteins present in the F508del-CFTR interactome

**Table S3 -** **GO analysis for differentially expressed proteins in the interactomes related to AFT recognition.** Biological process and cellular component are represented for each pair under comparison.

**Table S4 - GO analysis for protein clusters detected in PCA for full length CFTR variants.** Biological process terms are represented for each cluster detected.

**Table S5 - GO analysis for protein clusters detected in PCA for the protein sets corresponding to the arginine-framed tripeptides related checkpoint.** Biological process terms are represented for each cluster detected.

**Datasets**

**Dataset S1A** – Full list of interacting proteins for DD/AA-CFTR

**Dataset S1B** – Full list of interacting proteins for WT-CFTR

**Dataset S2A** – Proteins with higher affinity for DD/AA-CFTR

**Dataset S2B** – Proteins with higher affinity for WT-CFTR

**Dataset S3** – Proteins pulled down with peptides

**Dataset S4A** – Proteins with higher association to peptides R

**Dataset S4B** – Proteins with higher association to peptides K

**Dataset S5** – Lists of common proteins for Jaccard similarity test

**Dataset S6** – Lists of hits for volcano plots

**Dataset S7** – Proteins present in F508del-CFTR interactome but not WT- not DD/AA-CFTR
