# Supplementary figures and images for: Folding Status Is Determinant over Traffic-Competence in Defining CFTR Interactors in the Endoplasmic Reticulum
Source: Cells. 2019 Apr 14;8(4):353. doi: 10.3390/cells8040353 (PMC6523853; doi:10.3390/cells8040353)

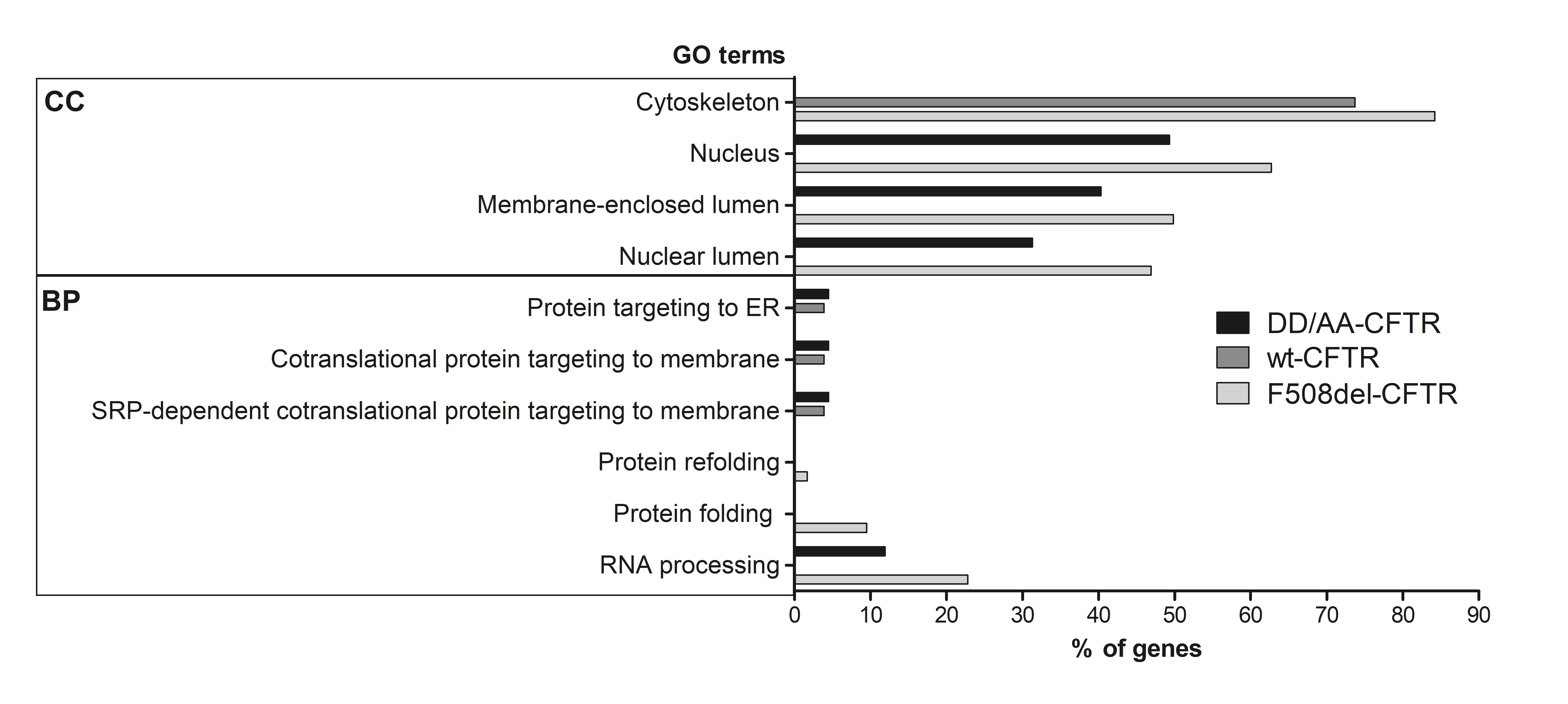

Supplement: Supplementary file 1 [file cells-08-00353-s001.zip › Supplementary materials/FigS4.jpg]

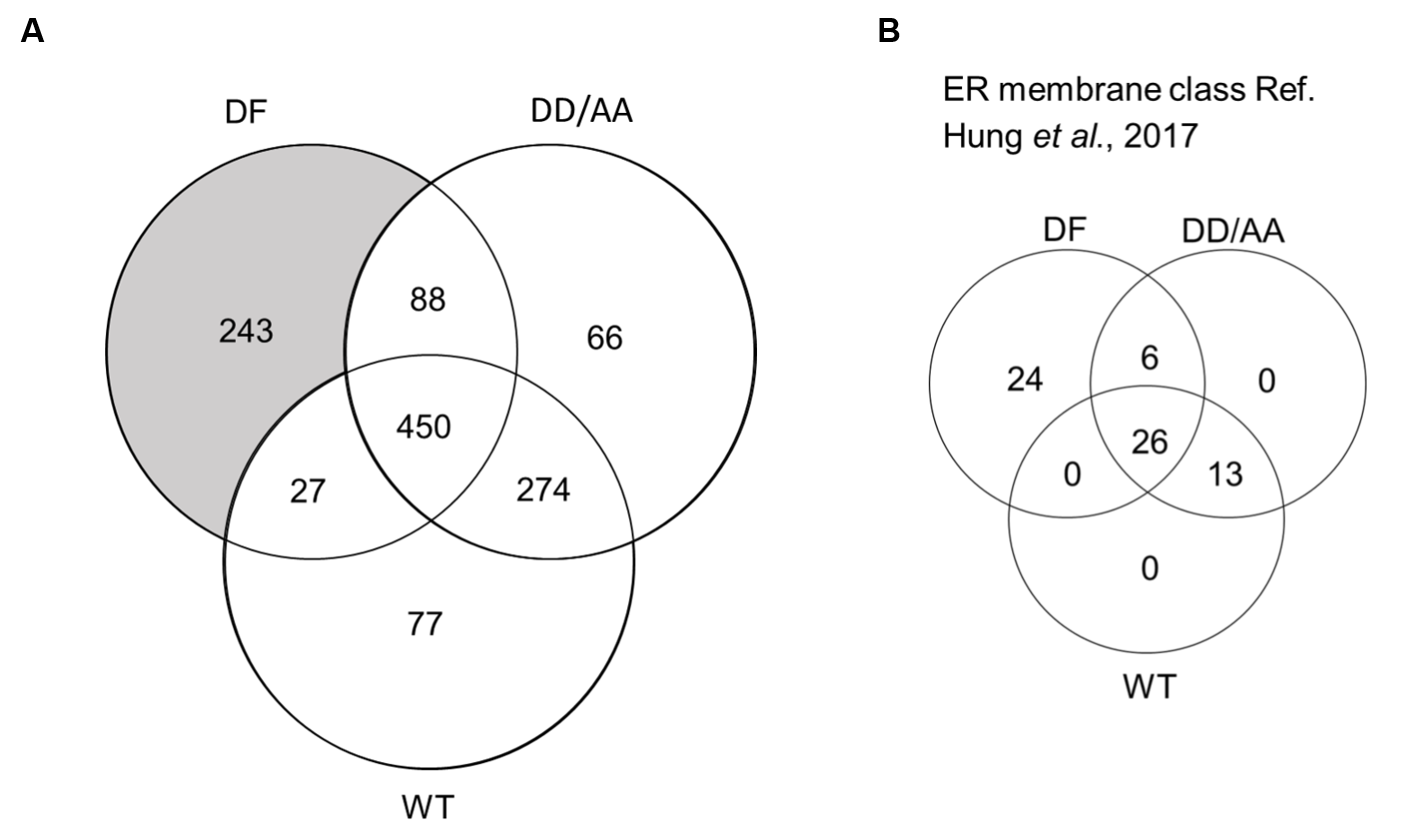

Supplement: Supplementary file 1 [file cells-08-00353-s001.zip › Supplementary materials/FigS3.tif]

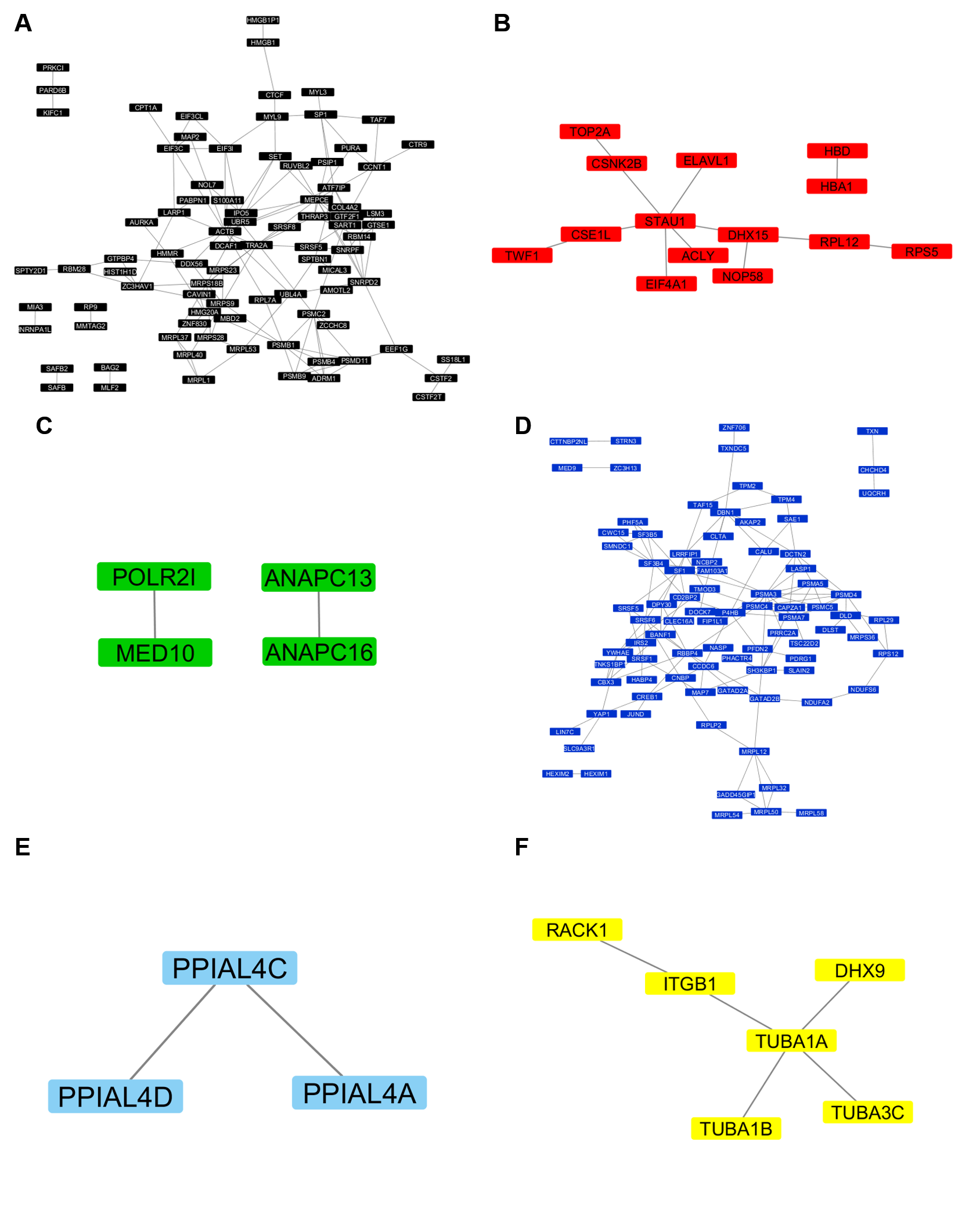

Supplement: Supplementary file 1 [file cells-08-00353-s001.zip › Supplementary materials/FigS6.tif]

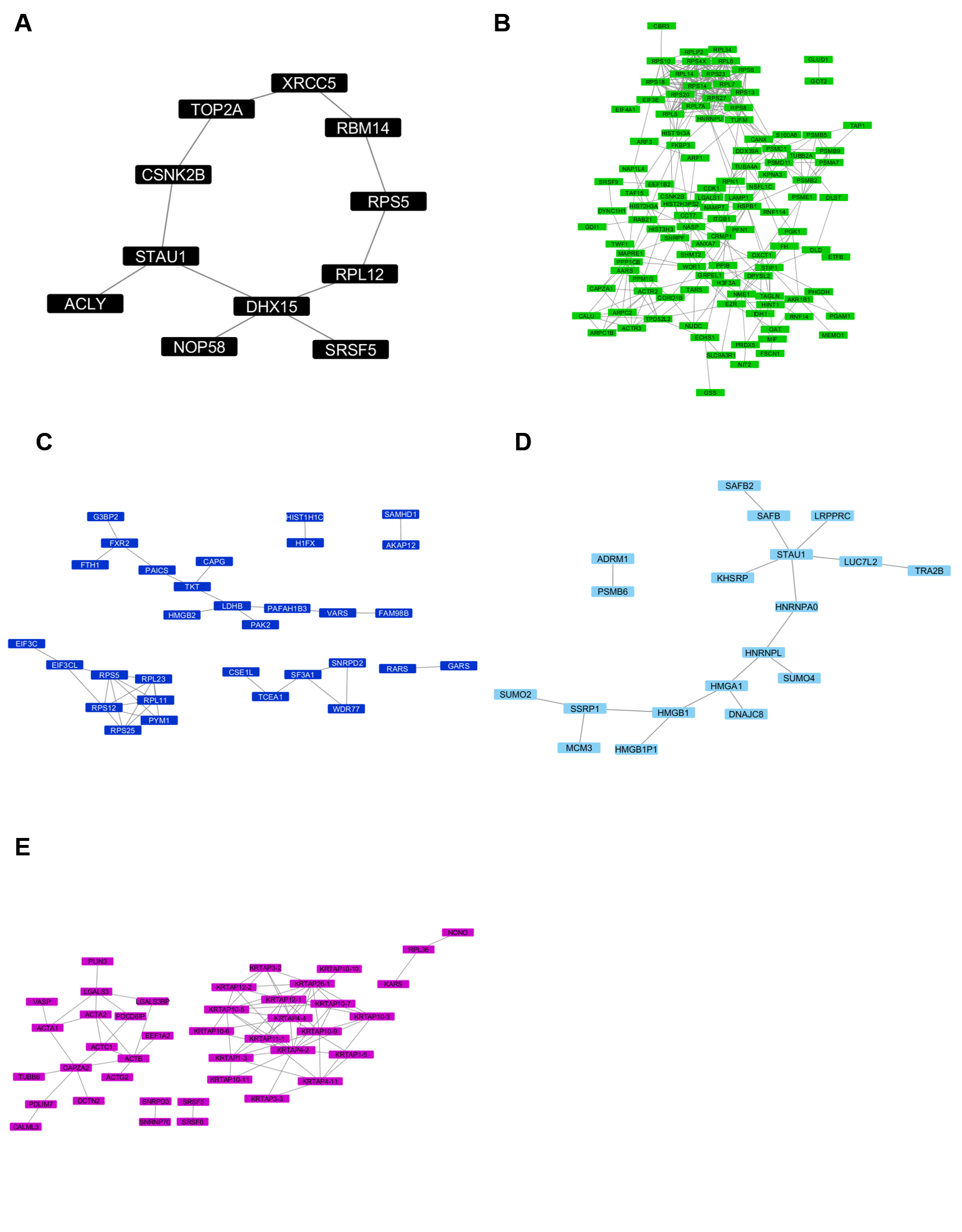

Supplement: Supplementary file 1 [file cells-08-00353-s001.zip › Supplementary materials/FigS5.tif]

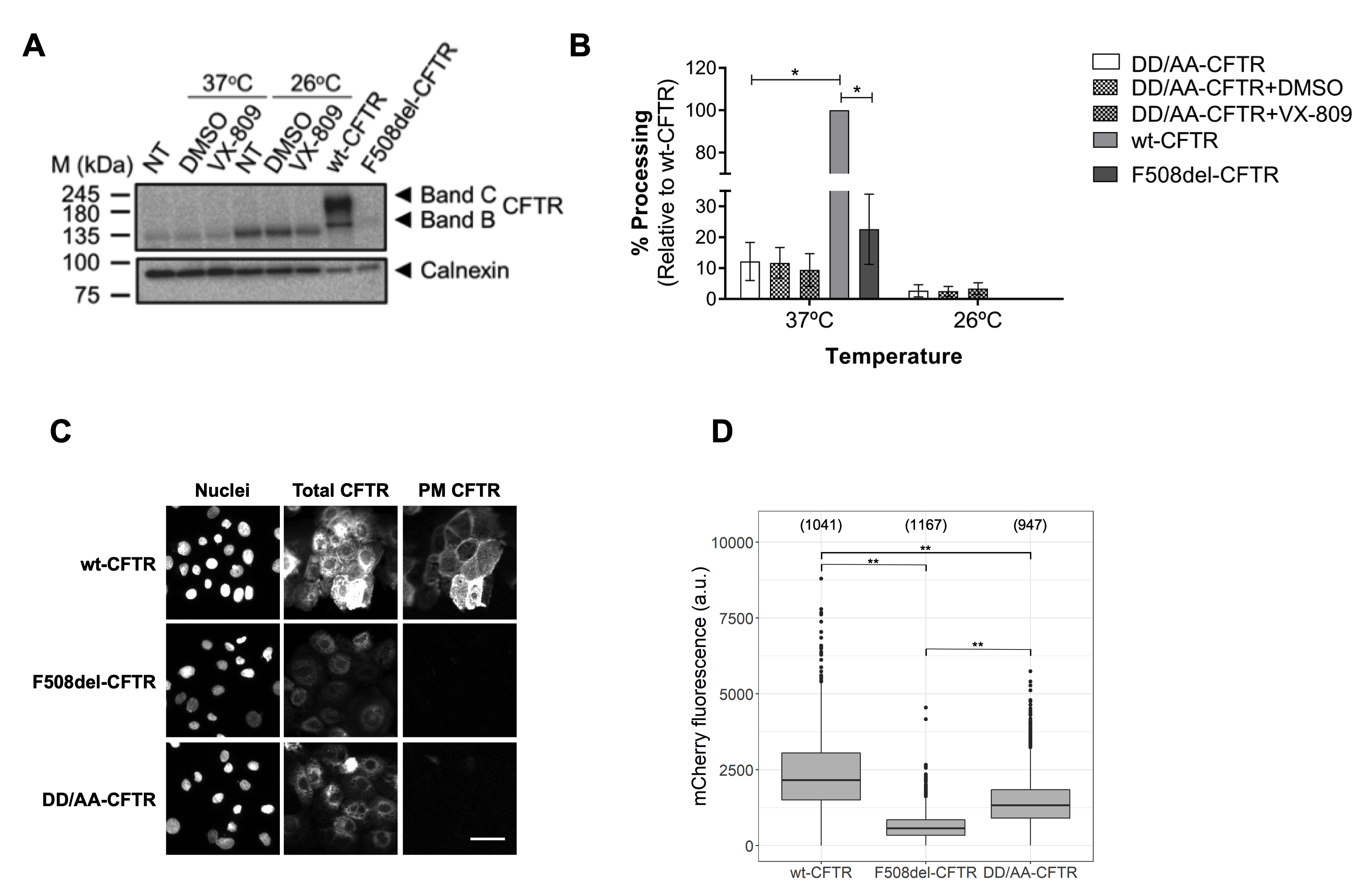

Supplement: Supplementary file 1 [file cells-08-00353-s001.zip › Supplementary materials/FigS1.tiff]
